# Cardiac remodeling and myocardial dysfunction in obese spontaneously hypertensive rats

**DOI:** 10.1186/1479-5876-10-187

**Published:** 2012-09-10

**Authors:** Dominik Linz, Mathias Hohl, Felix Mahfoud, Jan-Christian Reil, Wolfgang Linz, Thomas Hübschle, Hans-Paul Juretschke, Claudia Neumann-Häflin, Hartmut Rütten, Michael Böhm

**Affiliations:** 1 Klinik für Innere Medizin III, Kardiologie, Angiologie und Internistische Intensivmedizin, Universitätsklinikum des Saarlandes, Homburg/Saar, 66421, Germany; 2 Sanofi-Aventis, Diabetes Division and BioImaging, Frankfurt, Germany

**Keywords:** SHR-ob, SHR, MRI, Metabolic syndrome, Hypertension, Remodeling

## Abstract

**Background:**

The additive effects of obesity and metabolic syndrome on left ventricular (LV) maladaptive remodeling and function in hypertension are not characterized.

**Methods:**

We compared an obese spontaneously hypertensive rat model (SHR-ob) with lean spontaneously hypertensive rats (SHR-lean) and normotensive controls (Ctr). LV-function was investigated by cardiac magnetic resonance imaging and invasive LV-pressure measurements. LV-interstitial fibrosis was quantified and protein levels of phospholamban (PLB), Serca2a and glucose transporters (GLUT1 and GLUT4) were determined by immunohistochemistry.

**Results:**

Systolic blood pressure was similar in SHR-lean and SHR-ob (252 ± 7 vs. 242 ± 7 mmHg, p = 0.398) but was higher when compared to Ctr (155 ± 2 mmHg, p < 0.01 for both). Compared to SHR-lean and Ctr, SHR-ob showed impaired glucose tolerance and increased body-weight. In SHR-ob, LV-ejection fraction was impaired vs. Ctr (46.2 ± 1.1 vs. 59.6 ± 1.9%, p = 0.007). LV-enddiastolic pressure was more increased in SHR-ob than in SHR-lean (21.5 ± 4.1 vs. 5.9 ± 0.81 mmHg, p = 0.0002) when compared to Ctr (4.3 ± 1.1 mmHg, p < 0.0001 for both), respectively. Increased LV-fibrosis together with increased myocyte diameters and ANF gene expression in SHR-ob were associated with increased GLUT1-protein levels in SHR-ob suggestive for an upregulation of the GLUT1/ANF-axis. Serca2a-protein levels were decreased in SHR-lean but not altered in SHR-ob compared to Ctr. PLB-phosphorylation was not altered.

**Conclusion:**

In addition to hypertension alone, metabolic syndrome and obesity adds to the myocardial phenotype by aggravating diastolic dysfunction and a progression towards systolic dysfunction. SHR-ob may be a useful model to develop new interventional and pharmacological treatment strategies for hypertensive heart disease and metabolic disorders.

## Introduction

Hypertension, obesity and metabolic syndrome are highly prevalent cardiovascular diseases and risk factors in industrialized countries [1]. Type II diabetes and obesity are common comorbidities in patients with hypertension [2-4] and increase all cause mortality [5-8]. Interestingly, metabolic syndrome amplifies cardiovascular risk associated with high blood pressure independent of the effect of several traditional cardiovascular risk factors in hypertensive subjects [9]. In hypertension, obesity and metabolic syndrome frequently coexist and increase the prevalence of heart failure and in particular heart failure with preserved ejection fraction and diastolic dysfunction [10,11].

The aim of the present study was to systematically characterize maladaptive remodeling processes (functional, histological and molecular) in an obese spontaneously hypertensive rat model carrying an additional mutation in the leptin receptor (SHR-ob) [12] compared tolean spontaneously hypertensive rats (SHR-lean) and normotensive controls (Ctr). The SHR-ob rat is an unique animal model expressing multiple abnormal phenotypes including obesity, hypertension, hyperinsulinemia and hyperlipidemia [12]. A detailed characterization of the cardiac phenotype of this rat model is lacking, however, important for future investigations of therapeutic interventions.

## Materials and methods

### Animals

Male obese spontaneously hypertensive rats (SHR-ob, n = 8), their heterozygous control littermates (SHR-lean, n = 8) and male normotensive Sprague Dawley (Ctr, n = 10), were purchased from Charles River Germany GmbH (Sulzfeld, Germany) at an age of ten weeks. We used Sprague Dawley rat as the correct and only normotensive control for SHR-ob rats, as they were developed from a cross between a SHR-lean and a normotensive Sprague Dawley rat (Charles River Germany GmbH). However, Sprague Dawley rat does not represent the most appropriate control for the SHR-lean rat. Therefore, some of the comparisons in this study need to be seen with precaution. The animals were housed individually in standard cages and received standard chow diet (standard diet #1320, Altromin, Lage, Germany) and tap water ad libitum. The animal experiments were conducted in accordance with the National Instructions oh Health (NIH) Guide for the Care and Use of Laboratory Animals and with the Welfare guidelines and the German law for the protection of animals. The study was approved by the regional commission on charge in Darmstadt, Germany.

Metabolic characterization was performed at age of 38 weeks. Blood was obtained from the retro-orbital plexus under light anesthesia (3.5% isoflurane). Blood glucose and glycated hemoglobin (HbA1c) were measured using standard kits (Cobas Integra, Roche diagnostics, Mannheim, Germany). An oral glucose tolerance testing was performed at 38 weeks consisting of glucose measures at baseline and at 15, 30, 60, 90, 120 and 180 minutes after oral glucose loading (2 g/kg body weight). Systolic blood pressure and heart rate were measured by tail cuff method in conscious rats. At 38 weeks of age, cardiac LV-function was assessed by cardiac magnetic resonance imaging (MRI). One week later, invasive LV-pressure measurements were performed and the animals were afterwards sacrificed.

### MRI Acquisition and analysis

At the age of 38 weeks, rats were anesthetized with 1.5-2.5% isoflurane. Rats were then positioned prone and horizontally in a custom-manufactured animal holder in the magnet and allowed to breath freely. All experiments were performed with a Bruker Biospec 70/30 (Bruker Biospin, Ettlingen, Germany) MRI system, operating at 300MHZ with a bore of 30 cm. The assembly used consisted of the gradient system BGA-12S and a rapid-QUAD-Birdcage (112/90) resonator. Scout images of the individual rat heart were measured in a coronal (4-chamber-view) and sagittal (2-chamber-view) orientation. Sixteen contingent slices were acquired in short-axis orientation covering the entire heart. To cover the cardiac cycle ten time frames for each slide of the heart were retrospectively reconstructed with the customized program IntraAngio within the BrukerVision 5.0 software. For analysis, boundaries of the LV were obtained in each short-axis image by manual tracing [13]. All dimensions were measured throughout the cardiac cycle. LV end-diastolic volume (LVedV) and end-systolic volume (LVesV) were derived from the volume-time curves for maximal and minimal values. Stroke volume (SV) and LV-ejection fraction (EF) were subsequently computed. Enddiastolic LV-mass was derived from the sum of the differences between the epicardial (EPI) and endocardial (ENDO) areas from apex to base, using the formula: LV-mass = ∑(EPI-ENDO)*(THK + GAP)*1.05, where THK is the slice thickness, GAP is the interslice gap, and 1.05 represents the density of myocardial tissue (g/cm3). LV-mass, LVedV and LVesV were normalized on individual tibia length/tibia length of SHR. Regional myocardial diastolic LV-wall thickness was assessed automatically in one slice at the level of papillary muscles. LV-wall was devided in 97 chords.

### Cardiac LV-pressure measurements

At age 39 weeks, the animals were anaesthetized with thiopental (Narcoren, 100 mg/kg i.p., Merial, Hallbergmoos, Germany), intubated and lungs were artificially ventilated. LV-pressures were assessed using a Millar Tip catheter (Millar Instruments Inc, Houston, USA), which was introduced from the right carotid artery and advanced into the LV-cavity. Data were digitized with a sampling rate of 1000 Hz and recorded on a PC using specialized software (HEM, Notocord, Croissy, France). LV peak systolic pressure (LVesP), maximal positive LV-pressure development (+dP/dt_max_), were determined as measurements of systolic LV-performance. Diastolic LV-functional parameters were: end diastolic pressure (LVedP), maximal LV-pressure decay (−dP/dt_max_) and the time constant of LV-pressure decay (Tau), calculated according to Weiss et al. [14]. After completion of hemodynamic measurements, the animals were sacrificed by quick excision of the hearts under continued deep anesthesia. The hearts were arrested in the diastolic state with intravenous potassium chloride injection and quickly excised. After rapid excision and careful removal of atrial and non-cardiac tissue, total heart weight and LV-heart weight were measured. Aliquots of the tissues were snap-frozen for RNA and protein analysis.

### Heart and pancreas tissue analysis

Part of the LV containing the papillary muscles level was fixed in buffered 4% paraformaldehyde for 24 hours and embedded in paraffin for histological evaluation. LV sections at the papillary muscles level were cut at 5 μm, deparaffinized, rehydrated and stained with Picro-Sirius Red to visualize interstitial and perivascular fibrosis. The percentage of the LV consisting of interstitial collagen was calculated as the ratio of Picro-Sirius-Red positively stained area over total LV tissue area using Morpho Expert image analysis software (Explora Nova, La Rochelle, France). A further set of LV sections were additionally stained with hematoxylin and eosin (H&E). 90–100 cells/animal were measured to determine cardiomyocyte diameter. Leica Qwin version 3 morphometry software (Leica, Cambridge, UK) was used for analysis.

In order to immunohistologically analyze the pancreatic ß-cell area, pancreas was immersion fixed in buffered 4% paraformaldehyde, embedded in paraffin, cut at 5 μm and stained with a Ventana Discovery XT autostainer (Roche Diagnostics GmbH, Mannheim, Germany) using a mouse monoclonal anti-insulin antibody (Abcam, ab6995, Cambridge, UK, 1:1000). The percentage of area stained for ß-cells was calculated as the ratio of insulin positively stained area over total pancreatic tissue area using Morpho Expert imaging analysis software (Explora Nova, La Rochelle, France).

### Gene expression and protein level analyses

Reverse Transcription: RNA was prepared from rat LV-tissues using peqGold TriFast (PeqLab, Erlangen, Germany) extraction reagent per manufacture′s protocol. For cDNA preparation 2 μg of RNA were digested with DNAse (Peqlab, Erlangen) than reverse transcribed using the HighCap cDNA RT Kit (Applied Biosystems, Darmstadt, Germany) according to the manufacture′s protocol.

TaqMan PCR: TaqMan PCR was conducted in a StepOne plus thermocycler (Applied Biosystems, Germany) using TaqMan GenEx Mastermix (Applied Biosystems, #4369016). Signals were normalized to corresponding glyceraldehyde-3-phosphate dehydrogenase (GAPDH) controls. No template controls were used to monitor for contaminating amplifications. The ΔCt was used for statistical analysis and 2^−ΔΔCt^ for data presentation. Probes used to amplify the transcripts were as follows (purchased by Applied Biosystems): Nppa (RN00561661_m1); Nppb (Rn00676450_g1); CTGF (Rn00573960_g1); TGFβ1 (Rn99999016_m1); Col1a (Rn01463848_m1); GAPDH (Rn99999916_s1).

Western blot analysis: Rat LV tissue was minced in liquid nitrogen and resuspended in 5 volumes of homogenization buffer (in mmol/L): Na_2_EDTA 5.0, NaF 25.0, sucrose 300.0, PMSF 1.0, benzamidine 1.0 and KH_2_PO_4_ 30.0 (pH 7.0) containing complete protease inhibitors (Roche, Germany) and phosphatase inhibitors (Roche, Germany). The homogenate was centrifuged at 16.000 g for 20 min. 50 μg of protein was separated on 8-12% SDS-PAGE and electrophoretically transferred to nitrocellulose membrane (0.2 μm pore size, Schleicher and Schuell, Dassel, Germany). Membranes were blocked in Tris-buffered saline (TBS) containing 5% nonfat dry milk for 120 min at room temperature and exposed to rabbit polyclonal anti-phospho-phospholamban Thr17 (sc-17024-R, Santa Cruz Biotechnology, CA, USA; dilution: 1:400), rabbit polyclonal anti-phospho-phospholamban Ser16 (#07-052, Upstate, Millipore: dilution: 1:400), mouse monoclonal anti-Phospholamban (#05-205, Millipore: dilution: 1:500), anti-goat polyclonal Serca2 (N-19, sc-8095, Santa Cruz, 1:1000), anti-mouse Glut4 (Cell Signaling; #2213S, 1:1000), anti-rabbit Glut1 (Millipore; #07-1401, 1:1000) and mouse monoclonal IgG GAPDH (6C5: sc-32233, Santa Cruz Biotechnology, 1:5000). Secondary antibodies goat anti-rabbit (Sigma-Aldrich, Deisenhofen, Germany; 1:2500), goat anti-mouse (170–6516 Bio-Rad, Germany; 1:2500) and rabbit anti-goat (#172-1034, Bio Rad, Germany; 1:5000) were incubated for 60 min at room temperature. Proteins were visualized by enhanced chemiluminescence according to the manufacturer′s guidelines (Amersham Pharmacia Biotech, Freiburg, Germany).

Membrane stripping and re-probing conditions: Membrane was stripped two times for 15 minutes at 56°C using stripping buffer (62.5 mM Tris–HCl (pH 6.8), 2% SDS. 280 μl of 2-beta mercaptoethanol or a final concentration of 0.1 M 2-mercaptoethanol), then thoroughly washed with 1xPBS (in mmol/L: NaCl 170, KCl 33, Na_2_HPO_4_ 40 and KH_2_PO_4_ 18, pH 7.2) for at least one hour and blocked again in Tris-buffered saline (TBS) containing 5% nonfat dry milk for 120 min at room temperature.

Autoradiographs were quantified by imaging densitometry and analyzed by the “ImageQuant-TM” b Software (Image Quant, Molecular Dynamics, Krefeld, Germany). Data are presented as intensity optical density (IOD). There were no differences between groups in control genes and proteins.

### Statistics

Data are presented as mean ± SEM and differences were tested for significance using an unpaired Student’s *t*-test or 2-way-ANOVA with Bonferroni post-hoc test when appropriate. A p-value of less than 0.05 was considered statistically significant.

## Results

### General characteristics

**Figure 1:**
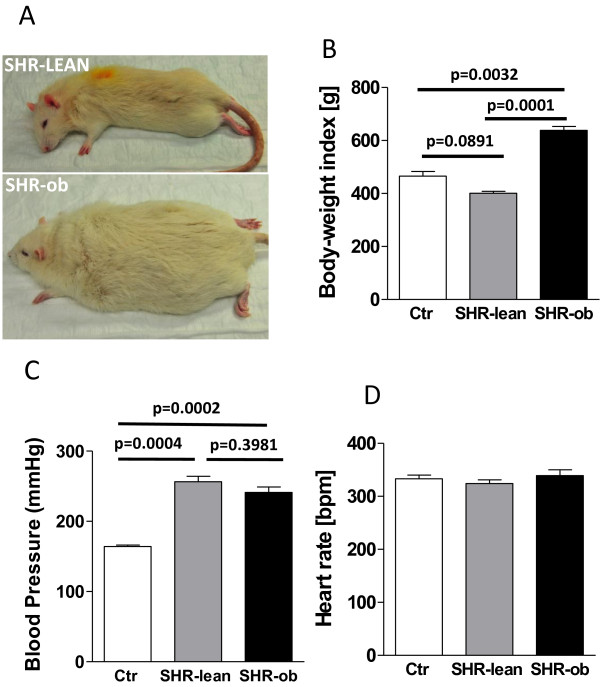
**A) Phenotypes of SHR-lean and SHR-ob rat are shown for comparison.** Quantification of **B)** body-weight index (body-weight normalized on individual tibia length/tibia length of SHR-ratio), **C)** systolic blood pressure **D)** and heart rate determined by tail cuff in controls (Ctr) (n = 10), SHR-lean (n = 8) and SHR-ob (n = 8) at 38 weeks of age. Data are presented in means ± SEM.

In Figure 1A, phenotypes of SHR-lean and SHR-ob rats are shown for comparison. At 38 weeks of age, body-weight index (body-weight normalized on individual tibia length/tibia length of SHR-ratio) was significantly higher compared to SHR-lean and Ctr (Figure 1B). Systolic blood pressure determined by tail cuff in awake rats was similar in SHR-lean and SHR-ob and significantly increased when compared with normotensive controls (Figure 1C). Heart rate did not differ between Ctr, SHR-lean and SHR-ob (Figure 1D). Wet lung weight did not differ between the groups (1610 ± 15 mg in Ctr, 1669 ± 21 mg in SHR-lean, 1659 ± 23 mg in SHR-ob, normalized on individual tibia length/tibia length of SHR-ratio, n.s.) suggesting a compensated state of congestion and heart failure.

**Figure 2:**
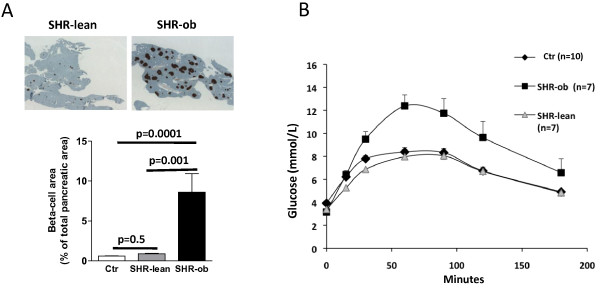
**A) Beta islet cell detection and quantification in pancreatic tissue via insulin immunohistochemistry (pancreatic beta-cell areas of total pancreatic area) in controls (Ctr), SHR-lean and SHR-ob rats. B)** Oral glucose tolerance testing in controls (Ctr) (n = 10), SHR-lean (n = 7) and SHR-ob (n = 7) for comparison. Data are presented in means ± SEM.

Metabolic parameters are summarized in Table 1. Fasting serum insulin levels were significantly elevated in SHR-ob compared with SHR-lean and Ctr. However, SHR-ob did not show fasting hyperglycemia and HbA1c was not increased. SHR-ob showed increased cholesterol and triglyceride blood-levels compared to SHR-lean and controls. Increased insulin levels in SHR-ob were associated with increased beta islet cell per total pancreatic area compared to SHR (Figure 2A). In response to glucose challenge, the SHR-ob had a more sustained increase in plasma glucose, with significantly higher glucose values at 60, 90 and 120 min compared with SHR-lean and controls, indicating a glucose intolerance in SHR-ob (Figure 2B).

**Table 1:** Metabolic parameters of Ctr, SHR-lean and SHR-ob at 38 weeks of age

	**Controls (1)**	**SHR-lean (2)**	**SHR-ob (3)**	**p-values**		
	**(n = 10)**	**(n = 8)**	**(n = 8)**	**(1) vs. (2)**	**(1) vs. (3)**	**(2) vs. (3)**
**Fasting glucose [mmol/L]**	6.6 ± 0.2	5.4 ± 0.2	6.2 ± 0.3	0.0412	0.2231	0.1129
**Insulin [μLg/dl]**	1.2 ± 0.2	0.91 ± 0.13	64.3 ± 15.9	0.6692	0.0001	0.0029
**HbA1c [%]**	4.0 ± 0.1	3.9 ± 0.0	4.2 ± 0.2	0.4419	0.3056	0.3702
**Cholesterol [mmol/L]**	1.4 ± 0.1	2.4 ± 0.1	10.3 ± 1.4	0.0061	0.0001	0.0002
**Triglyceride [mmol/L]**	1.2 ± 0.2	1.3 ± 0.1	6.65 ± 1.61	0.0792	0.0004	0.0001

Data are presented in means ± SEM.

### LV-hemodynamics

Cardiac MRI at the age of 38 weeks revealed lower LV-ejection fraction in SHR-ob rats compared to controls and SHR-lean (Table 2). Cardiac index was reduced in SHR-ob compared to Ctr and SHR-lean mainly due to reduced stroke volume as heart rate did not differ significant between the groups. Enddiastolic LV volume was increased, while enddiastolic wall thickness was unchanged (not shown).

**Table 2:** LV-function determined by cardiac magnetic resonance imaging

	**Controls (1)**	**SHR-lean (2)**	**SHR-ob (3)**	**p-values**		
	**(n = 10)**	**(n = 8)**	**(n = 8)**	**(1) vs. (2)**	**(1) vs. (3)**	**(2) vs. (3)**
**Heart rate [1/min]**	359 ± 13	354 ± 8	346 ± 12	0.2722	0.0710	0.1824
**Ejection fraction [%]**	59.6 ± 3.3	54.4 ± 2.4	46.2 ± 3.9	0.0722	0.0071	0.1824
**ED volume index [μL]**	636 ± 26	620 ± 20	651 ± 29	0.0823	0.0629	0.0332
**ES volume index [μL]**	257 ± 15	282 ± 9	353 ± 35	0.0487	0.0002	0.0092
**Stroke volume index [μL]**	379 ± 19	338 ± 16	298 ± 22	0.0792	0.0009	0.0029
**Cardiac index [ml/min]**	135 ± 12	120 ± 8	103 ± 13	0.3315	0.0021	0.0291

Data are presented in means ± SEM: *LV*: left ventricular, bpm: beats per minute; *ED*: enddiastolic; *ES*: endsystolic.

ED and ES Volume, Stroke volume was normalized on individual tibia length/tibia length of SHR-ratio (Index).

**Figure 3:**
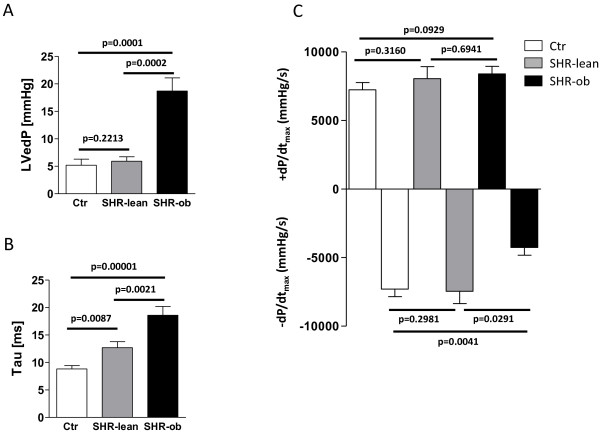
**LV-function determined by invasive LV-measurement procedures: A) Left ventricular enddiastolic pressure (LVedP), B) left ventricular isovolumetric relaxation time constant (Tau), C) maximal (+dP/dt**_**max**_**) and minimal (−dP/dt**_**max**_**) pressure decay in controls (Ctr) (n = 10), SHR-lean (n = 8) and SHR-ob (n = 8).** Data are presented in means ± SEM.

During invasive LV-pressure measurement, LVesP was significantly increased in SHR-lean and SHR-ob compared to controls to a similar extent (178 ± 11 mmHg and 183 ± 8 mmHg vs. 102 ± 4 mmHg, p < 0.0001 for both). Indices of diastolic dysfunction (LVedP and Tau, Figure 3A-B) were significantly higher in SHR-ob compared to normotensive controls and SHR-lean. Maximal positive LV-pressure development (+dP/dt_max_) in all groups was unchanged. However, maximal pressure decay during diastole (−dP/dt_max_) was impaired in SHR-ob but unchanged in SHR-lean when compared to normotensive controls (Figure 3C).

Estimated LV capacitance of SHR-ob was decreased indicated by a leftwards shift of the LVedP/LVedV-diagram and an increased LVedP/LVedV-ratio due to higher enddiastolic pressures and moderate increases in enddiastolic volumes in SHR-ob compared to controls (0.051 ± 0.008 mmHg/μL in SHR-ob vs. 0.006 ± 0.001 mmHg/μL in Ctr, p < 0.0001). Estimated LV capacitance of SHR-lean was not changed compared to Ctr (0.011 ± 0.003 mmHg/μL, p > 0.05 vs. Ctr).

### Cardiac structural-remodeling and differential gene expression

**Figure 4:**
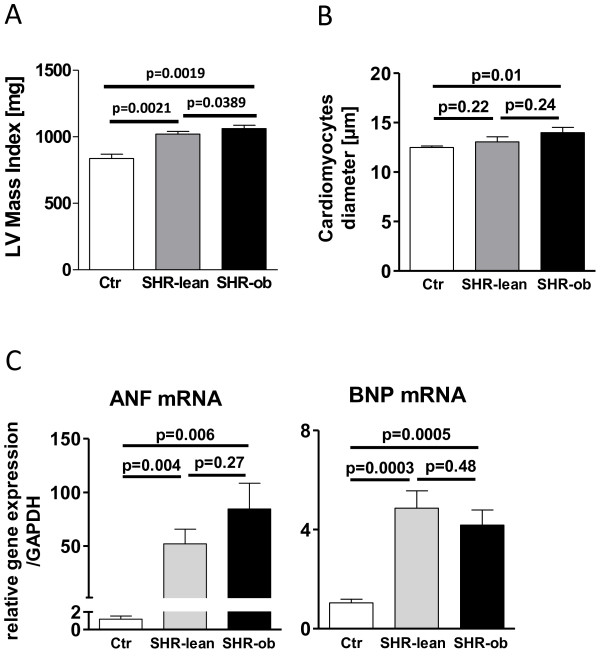
**A) Left ventricular mass, normalized on individual tibia length/tibia length of SHR-ratio (LV Mass index), B) cardiomyocyte diameter, left ventricular gene expression of C) ANF and D) BNP in controls (Ctr), SHR-lean and SHR-ob (n = 6 each).** GAPDH was used as loading control. Data are presented in means ± SEM.

**Figure 5:**
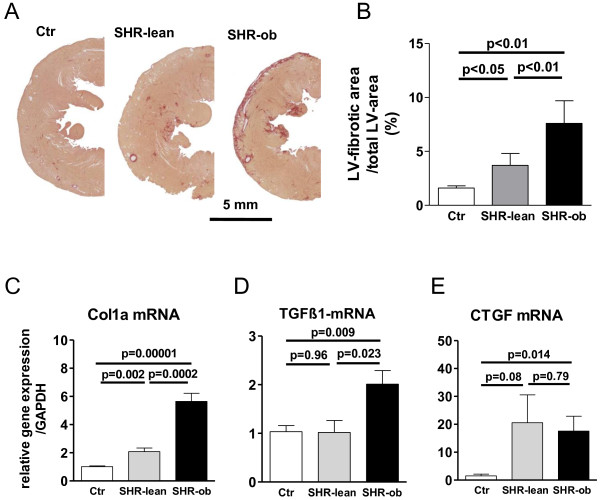
**A) Representative histological pictures and B) quantification of left ventricular fibrotic area in controls (Ctr), SHR-lean and SHR-ob.** Sirius red was used to stain collagen. Gene expression of **C)** Col1a, **D)** TGFβ1 and **E)** CTGF mRNA in controls (Ctr), SHR-lean and SHR-ob, normalized to GAPDH expression (n = 6 each). Data are presented in means ± SEM.

In SHR-ob, increased LV-mass indices and cardiomyocyte diameters were associated with up-regulation of BNP and ANF gene expression (Figure 4A-D). Picro-Sirius Red staining demonstrated an increase in interstitial collagen deposition in SHR-lean and in SHR-ob compared to controls (Figure 5A-B). SHR-ob showed the highest amount of interstitial fibrosis. The papillary muscle was a prominent fibrotic site. In the left ventricle, mRNA levels of collagen1a (Col1a) (Figure 5C), transforming growth factor beta 1 (TGFβ1) (Figure 5D) and connective tissue growth factor (CTGF) (Figure 5E) were significantly increased in SHR-ob compared to controls. By contrast, in SHR-lean, only Col1a was significantly increased compared to controls but significantly less pronounced compared to SHR-ob. TGFβ1 mRNA expression was unchanged in SHR-lean compared to controls. However, CTGF was increased in SHR-lean but failed to reach significance.

### Proteins involved in Ca^2+^-handling

**Figure 6:**
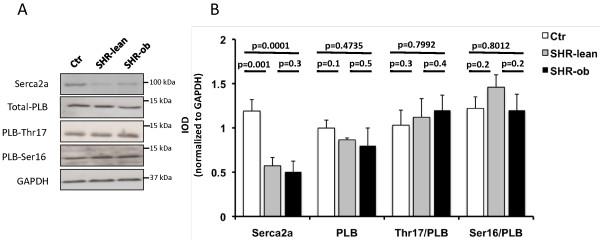
**A) Representative western blots and B) quantification of Serca2a, total phospholamban (PLB) and its phosphorylation at Threonine 17 and Serine 16 in controls (Ctr), SHR-lean and SHR-ob (n = 6-8).** GAPDH was used as loading control. Data are presented in means ± SEM. All remaining western blots are provided as online (Additional file 1: Figure S1).

Analyses of protein-levels involved in calcium-handling are shown in Figure 6 and Additional file 1: Figure S1 (Online Supplements). The protein abundance of Serca2a was significantly reduced in SHR-lean and SHR-ob compared to Ctr. Total phospholamban (PLB) protein was similar in all groups. PLB phosphorylation at serine 16 (Ser16-PLB) and at threonine 17 (Thr17-PLB) functionally enhances Serca2a activity and Ca^2+^ uptake in the SR. Ser16-PLB to total PLB ratios and Thr17-PLB to total PLB ratios was not modified in SHR-lean and SHR-ob.

### Glucose transporters

**Figure 7:**
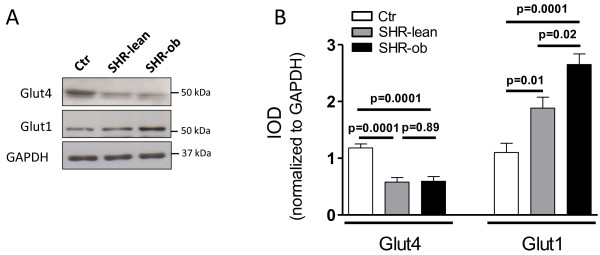
**A) Representative western blots and B) quantification of glucose transporters Glut4 and Glut1 in controls (Ctr), SHR-lean and SHR-ob (n = 6 each).** GAPDH was used as a loading control. Data are presented in means ± SEM.

GLUT4 protein amount was significantly reduced in LV-tissue of SHR-lean and SHR-ob compared to controls. In contrast GLUT1 protein amount was significantly increased in LV-tissue of SHR-lean and of SHR-ob. The increase in GLUT1 protein amount was more pronounced in SHR-ob compared to SHR-lean (Figure 7).

## Discussion

Obese spontaneously hypertensive rats (SHR-ob) exhibit hyperinsulinemia and demonstrate glucose intolerance in response to oral glucose load although they were not overtly diabetic according to fasting glucose and HbA1c-levels. Identification of a null mutation in the leptin receptor gene in the SHR-ob rats [12] suggests that these animals exhibit insulin resistance resulting from the absence of leptin receptors. Additionally, SHR-ob uniformly developed hyperlipidemia, which was characterized by a marked rise in plasma triglycerides and plasma cholesterol. The rat model of SHR-ob is suitable for investigating the add-on effect of obesity and metabolic syndrome in hypertension on maladaptive cardiac structural, metabolic and functional remodeling processes. Additionally SHR-ob may be a useful model to develop new interventional and pharmacological treatment strategies for hypertensive heart disease and metabolic disorders.

Obesity and metabolic syndrome combined with hypertension in SHR-ob but not hypertension alone in SHR-lean resulted in a moderate decrease in LV-systolic function when compared to normotensive controls but LV-systolic global contractility was preserved as indicated by LV-pressure measurements. Reduced maximal pressure decay during diastole together with increased LVedP, increased Tau and decreased LV- capacitance (increased LVedP/LVedV-ratio) suggest the development of diastolic dysfunction in SHR-ob. Therefore the combination of obesity and hypertension in SHR-ob aggravates diastolic dysfunction and a progression towards systolic dysfunction compared to hypertension alone in SHR-lean. In accordance with this finding, the combination of hypertension, obesity and metabolic syndrome increases prevalence of heart failure and in particular heart failure with preserved ejection fraction (diastolic dysfunction) and has been identified as a relevant risk factor for heart failure in humans [10,11].

Hearts of SHR-ob showed increased cardiomyocyte diameters and increased LV mass index. Additionally, increased enddiastolic LV volume together with unchanged enddiastolic wall thickness suggests cardiac hypertrophy. Cellular hypertrophy was associated with increased LV ANF-gene expression in SHR-ob reflecting the effect of increased afterload in this model [15]. Cardiac hypertrophy due to long-standing arterial hypertension is associated with a high incidence of heart failure in humans [16].

Metabolic syndrome and obesity combined with hypertension in SHR-ob resulted in increased interstitial collagen deposition compared to hypertension alone in SHR. Increased expression of Col1a suggests that alterations in the rate of collagen synthesis are responsible for increased fibrosis in SHR-ob. TGFβ1 has been reported to be involved in fibroblast proliferation and extracellular matrix deposition. Gene expression of TGFβ1 and CTGF was elevated in SHR-ob but not in SHR-lean and may be contributory to the subsequent pronounced cardiac fibrosis in SHR-ob [17]. CTGF is induced by TGFβ1 in connective tissue cells and may represent a downstream mediator of TGFβ1-activity [18]. Increased collagen deposition may be a relevant factor in the observed development of diastolic dysfunction in SHR-ob [19].

Impaired LV-relaxation has been related to diastolic Ca^2+^ overload and consequently prolonged interaction of myofilaments in hypertensive cardiomyopathy [20,21]. The predominant alteration in Ca^2+^ homeostasis is a decreased uptake of Ca^2+^ into the sarcoplasmic reticulum (SR) due to decreased Serca2a protein levels [22]. Decreased Serca2a protein levels occur already at the stage of mild-to-moderate cardiac hypertrophy, precedes LV-dysfunction and is closely related to the degree of cardiac hypertrophy [20]. In the present study, a reduction in Serca2a protein level was observed in hypertrophied LV myocardium of SHR-lean and SHR-ob. This may mediate reduced SR Ca^2+^ stores and slow Ca^2+^ transient decay. The reduction in the expression of cardiac Serca2a in SHR-lean and SHR-ob was accompanied by the development of diastolic dysfunction. PLB phosphorylation by protein kinase A (PKA) at serine 16 (Ser16-PLB) and by CaMKII at threonine 17 (Thr17-PLB) functionally enhances Serca2a Ca^2+^ uptake. However, Ser16-PLB to total PLB ratios and Thr17-PLB to total PLB were not significantly modified in SHR-lean and SHR-ob. This suggests that metabolic syndrome at the background of hypertension and hypertension alone causes a reduction in Serca2a protein levels, which may play a role for the development of diastolic dysfunction in SHR-lean and SHR-ob rats. Increased interstitial fibrosis amount and possibly also changes in different proteins involved in cardiac myocyte relaxation (e.g. titin) could account for the more pronounced diastolic dysfunction in SHR-ob compared to SHR-lean.

In SHR-ob, cardiac protein expression of GLUT1 was increased compared to SHR-lean and normotensive controls. During euglycemic hyperinsulinemia, the insulin independent glucose transporter GLUT1 is a major mediator of basal cardiac glucose uptake [23,24]. In diabetes and hypertension [25], there is profound downregulation of GLUT4. A lower GLUT4 content in hypertrophied hearts was associated with cardiac insulin resistance [25] but not with a reduction in basal glucose uptake [26] as deletion of GLUT4 is associated with induction of GLUT1 expression [27] compensating for the loss of function of GLUT4. Interestingly, fetal hearts have less GLUT4 and more GLUT1 than adult hearts [28]. Increased GLUT1 expression together with the induction of natriuretic peptide gene expression as one of the most conserved features of ventricular hypertrophy in SHR-ob suggests an upregulation of GLUT1/ANF-axis being in agreement with the concept of re-expression of fetal genes in cardiac hypertrophy. A comparable activation of the GLUT1/ANF-axis has already been described in pure diabetic [23] and hypertensive [24,25] rat models with cardiac hypertrophy.

The more pronounced structural and metabolic changes together with changes in proteins involved in calcium handling observed in our study may provide a sufficient explanation for the observed add-on effect of metabolic syndrome and obesity in hypertension on aggravation of diastolic dysfunction and a progression towards systolic dysfunction in SHR-ob compared to SHR-lean. Our findings in SHR-ob are in line with other rat models of hypertension combined with obesity and/or metabolic syndrome mainly characterized by cardiac hypertrophy and increased cardiac fibrosis [29,30]. This is the first report characterizing cardiac function and remodeling in SHR-ob rats.

## Conclusion and perspectives

We describe a rat model for maladaptive cardiac remodeling in obesity and metabolic syndrome associated with hypertension, a common comorbid condition, gaining increasing relevance. Obesity combined with hypertension further aggravates maladaptive remodeling leading to diastolic dysfunction and a progression towards systolic dysfunction mainly by a pronounced increase in LV-fibrosis, cellular hypertrophy and moderate changes in proteins involved in Ca^2+^-handling. Myocardial hypertrophy in obese hypertensive rats are associated with an increased upregulation of the cardiac GLUT1/ANF-axis. SHR-ob may be a useful model to further elucidate pathophysiological alterations to develop new interventional and pharmacological treatment strategies for hypertensive heart disease and metabolic disorders.

## Abbreviations

SHR-ob: Obese spontaneously hypertensive rats; SHR: Lean spontaneously hypertensive rats; Ctr: Controls; LV: Left ventricle; PLB: Phospholamban; GLUT: Glucose transporters; MRI: Magnetic resonance imaging; LVedV: LV enddiastolic volume; LVesV: LV endsystolic volume; LVedV: LV enddiastoliv pressure; LVesP: LV endsystolic pressure; dP/dtmax: Maximal LV-pressure development; Col1a: Collagen1a; TGFβ1: Transforming growth factor beta 1; CTGF: Connective tissue growth factor.

## Competing interests

The authors declare that they have no competing interests.

## Authors contributions

DL, MH, FM and JCR carried out the molecular genetic studies, invasive hemodynamic measurements and drafted the manuscript. TH carried out the immunoassays and histology. HPJ and CNH performed MRI scans. WL, HR and MB participated in the design of the study and performed the statistical analysis. All authors read and approved the final manuscript.

## Supplementary Material

Additional file 1 **Figure S1.** Western Blots analyses of protein expression involved in calcium handling. Western Blot analysis of Serca2a, total phospholamban (PLB) and its phosphorylation at Threonine 17 (Thr17) and Serine 16 (Ser 16) of controls (Ctr), SHR-lean and SHR-ob (n=6-8). GAPDH was used as loading control. In all experiments, Ctr, SHR-lean and SHR-ob tissues were compared by western blot analysis at the same time with identical conditions and separated on the same SDS-PAGE. Detection of phospholamban phosphorylation at Ser16 or Thr17 were performed on two separate gels as indicated. Membranes were stripped afterwards and analysed for total phospholamban and GAPDH as loading control. Background color of autoradiographs depended on different exposure times resulting from respective endogenous protein expression and concentration. Click here for file
