# Supplementary material for: Cardiac remodeling and myocardial dysfunction in obese spontaneously hypertensive rats
Source: J Transl Med. 2012 Sep 10;10:187. doi: 10.1186/1479-5876-10-187 (PMC3508805; doi:10.1186/1479-5876-10-187)
Supplement: Additional file 1 — Figure S1. Western Blots analyses of protein expression involved in calcium handling. Western Blot analysis of Serca2a, total phospholamban (PLB) and its phosphorylation at Threonine 17 (Thr17) and Serine 16 (Ser 16) of controls (Ctr), SHR-lean and SHR-ob (n=6-8). GAPDH was used as loading control. In all experiments, Ctr, SHR-lean and SHR-ob tissues were compared by western blot analysis at the same time with identical conditions and separated on the same SDS-PAGE. Detection of phospholamban phosphorylation at Ser16 or Thr17 were performed on two separate gels as indicated. Membranes were stripped afterwards and analysed for total phospholamban and GAPDH as loading control. Background color of autoradiographs depended on different exposure times resulting from respective endogenous protein expression and concentration. [file 1479-5876-10-187-S1.doc]

**Supplemental Figures**

Figure S1:

**Figure S1: Western Blots analyses of protein expression involved in calcium handling.**

Western Blot analysis of Serca2a, total phospholamban (PLB) and its phosphorylation at Threonine 17 (Thr17) and Serine 16 (Ser 16) of controls (Ctr), SHR-lean and SHR-ob (n=6-8). GAPDH was used as loading control.

In all experiments, Ctr, SHR-lean and SHR-ob tissues were compared by western blot analysis at the same time with identical conditions and separated on the same SDS-PAGE. Detection of phospholamban phosphorylation at Ser16 or Thr17 were performed on two separate gels as indicated. Membranes were stripped afterwards and analysed for total phospholamban and GAPDH as loading control. Background color of autoradiographs depended on different exposure times resulting from respective endogenous protein expression and concentration.
